# Primary versus Redo Urethroplasty: Results from a Single-Center Comparative Analysis

**DOI:** 10.1155/2020/7214718

**Published:** 2020-01-31

**Authors:** Wesley Verla, Marjan Waterloos, Anne-Françoise Spinoit, Sarah Buelens, Elise De Bleser, Willem Oosterlinck, Francisco Martins, Enzo Palminteri, Achilles Ploumidis, Nicolaas Lumen

**Affiliations:** ^1^Department of Urology, Ghent University Hospital, C. Heymanslaan 10, 9000 Ghent, Belgium; ^2^Departamento de Urologia, Universidade de Lisboa, Hospital de Santa Maria, Lisboa, Portugal; ^3^Center for Urethral and Genitalia Reconstructive Surgery, Arezzo, Italy; ^4^Department of Urology, Clinica Cellini, Humanitas Institute, Torino, Italy; ^5^Department of Urology, Athens Medical Centre, Athens, Greece

## Abstract

**Objectives:**

To explore the differences between primary and redo urethroplasty and to directly compare according stricture-free survival (SFS). *Materials and Methods*. Data of all male patients who underwent urethroplasty at Ghent University Hospital were collected between 2000 and 2018. Exclusion criteria for this analysis were age <18 years and follow-up <1 year. Two patient groups were created for further comparison: the primary urethroplasty (PU) group (no previous urethroplasty) and redo urethroplasty (RU) group (≥1 previous urethroplasty), irrespective of prior endoscopic treatments. A comparison between groups was performed using the Mann–Whitney *U* test and Fisher's Exact test. SFS was calculated using Kaplan–Meier statistics. A functional definition of failure, being the need for further urethral manipulation, was used. Uni- and multivariate Cox regression analyses were performed on the entire patient cohort.

**Results:**

805 patients were included. Median (IQR) follow-up of the PU (*n* = 556) and RU (*n* = 556) and RU (*p*=0.1). The RU group involved more penile strictures (*p*=0.1). The RU group involved more penile strictures (*p*=0.1). The RU group involved more penile strictures (*p*=0.1). The RU group involved more penile strictures (*p*=0.1). The RU group involved more penile strictures (*p*=0.1). The RU group involved more penile strictures (*p*=0.1). The RU group involved more penile strictures (*p*=0.1). The RU group involved more penile strictures (*p*=0.1). The RU group involved more penile strictures (

**Conclusions:**

Several differences between primary and redo urethroplasties exist. Redo urethroplasty entails a distinct patient population to treat and is, in general, associated with lower stricture-free survival than primary urethroplasty, although more homogeneous series are required to corroborate these results. Prior urethroplasty and diabetes are independent risk factors for urethroplasty failure.

## 1. Introduction

Urethroplasty is considered the standard treatment option for urethral stricture disease (USD) as it offers substantially higher long-term success rates than direct vision internal urethrotomy (DVIU) or urethral dilatation [1, 2]. However, despite its satisfying outcome, there is a subgroup of patients in which failure is encountered. Several risk factors for failure after urethroplasty have been described, among which prior therapy for USD [3–6]. Against this background, the question whether redo urethroplasty provides the same satisfying outcome as primary urethroplasty should be considered.

So far, scarce data are available on the management of recurrent USD and only little is known about the differences between primary and redo urethroplasty. A redo urethroplasty is often more challenging as recurrent urethral strictures usually have denser and more extensive scar tissue. Moreover, since potential grafts or flaps may have already been used in previous urethroplasties, the armamentarium of the surgeon becomes smaller in a redo setting [7].

Be that as it may, the prognostic significance of prior urethroplasty remains highly controversial since several authors have published their experience with redo urethroplasty and reported success rates equivalent to primary urethroplasty [5, 7–14]. However, these reports contain several limitations and the different definitions of failure and different follow-up protocols make it hard to draw adequate conclusions [15]. To date, the largest comparative series was published by Levine et al., but included only 49 redo procedures and despite the differences in stricture length, stricture location, and applied surgical technique between groups, a Cox regression analysis to confirm the prognostic value of these characteristics was not performed [11]. Undoubtedly, additional evidence upon this subject is required and should be based on a comparative analysis with a higher volume of redo urethroplasties.

Considering the above, the aim of this study is to explore the differences between primary and redo urethroplasty and to directly compare the according stricture-free survival (SFS), with more power than the existing reports.

## 2. Patients and Methods

### 2.1. Patients

A database of all male patients who underwent urethroplasty at Ghent University Hospital was enrolled between 2000 and 2018. This database contains extensive information about patient and stricture characteristics, previous interventions, and other relevant information (suprapubic catheter, urinary tract infection (UTI)). Exclusion criteria for this analysis were age <18 years and duration of follow-up <1 year. Within the included patients, two patient groups were created for further comparison: the primary urethroplasty (PU) group, defined as patients without previous urethroplasty, and the redo urethroplasty (RU) group, defined as patients who underwent ≥1 urethroplasty, both irrespective of prior DVIU/dilatation. The study was approved by the local Ethics Committee (EC/2014/0438) and all included patients provided written informed consent.

### 2.2. Perioperative Management

Preoperatively, patients were evaluated through history taking, physical examination, and technical investigations (uroflowmetry, ultrasonic residue measurement and retrograde urethrography (RUG) and/or voiding cystourethrography (VCUG) and/or cystoscopy). A urine culture was performed one week before urethroplasty and appropriate antibiotics were started 24 hours before surgery in case of infection. All operations were performed by two surgeons (W. O. and N. L.).

Generally, a VCUG was performed fourteen days postoperatively and in case of no extravasation of contrast, the transurethral catheter was removed. In cases with contrast extravasation, the transurethral catheter was replaced and a VCUG was again performed one week later.

Follow-up visits included history taking, physical examination, and uroflowmetry and were performed after three months, after one year, and annually thereafter. Additional technical investigations were only administered in case of arguments for urethroplasty failure such as symptoms or an obstructive voiding curve (<15 ml/s). A subgroup of patients was followed by the referring urologist due to practical considerations.

### 2.3. Statistical Analysis

Baseline and per- and postoperative characteristics were analyzed using descriptive statistics. The comparison between groups was performed using the Mann–Whitney *U* test and Fisher's Exact test for continuous and categorical variables, respectively. Complications within 90 days postoperatively were categorized according to the Clavien–Dindo classification system [16]. For SFS, the time-to-event was measured as the interval between the operation date and the date of the diagnosis of the failure. A functional definition of failure, being the need for further urethral manipulation (including simple dilatation), was used [17]. Patients were censored at the time of the latest follow-up or death. SFS was calculated using Kaplan–Meier statistics and groups were compared using the Log-Rank test. Uni- and multivariate Cox regression analyses with the calculation of the Hazard Ratio (HR) to predict failure were performed on the entire patient cohort for the following variables: age, stricture length, location, etiology, previous interventions, urethroplasty technique, comorbidities, presence of suprapubic catheter, and UTI. Only the statistically significant variables from the univariate analysis were entered in the multivariate analysis. All statistical tests were 2-sided and a *p* value <0.05 was considered statistically significant. The analysis was performed using SPSS® 25.0.

## 3. Results

In total, 805 patients were included in this study. The median follow-up of the PU group (*n* = 556) and RU group (*n* = 249) was 87 and 76 months, respectively (*p*=0.1). Baseline characteristics are displayed in Table 1. Age, follow-up, stricture length, and comorbidities were comparable between groups. Penile strictures (*p* < 0.001), strictures due to Lichen Sclerosus (*p*=0.016), and strictures due to failed hypospadias repair (*p*=0.004) were significantly more frequent in the RU group. RU techniques comprised significantly more multistage procedures (*p* < 0.001) and definitive perineal urethrostomies (*p*=0.001) and significantly less anastomotic repairs (*p* < 0.001) and free graft urethroplasties (*p*=0.028). In both groups, “other urethroplasty techniques” mainly consisted of meatoplasties (>95%), which were proportionally more frequently performed in the RU group (*p*=0.004).

Per- and postoperative characteristics are displayed in Table 2. The hospital stay was significantly longer in the PU group (*p*=0.01), in contrast to the comparable operation time, catheter stay and extravasation ratio. The complication rate was 25% and 24% for the PU and RU groups, respectively. In both groups, complications were predominantly low-grade (Clavien–Dindo grade 1-2: 175/805; 22%) [16]. Grade 3 complications involved urinary retention with placement of a suprapubic catheter (3/805; 0.37%) and hematomas (5/805; 0.62%), abscesses (5/805; 0.62%), fistulas (13/805; 1.6%), and Fournier gangrene (1/805; 0.12%) requiring surgical intervention.

**Figure 1:**
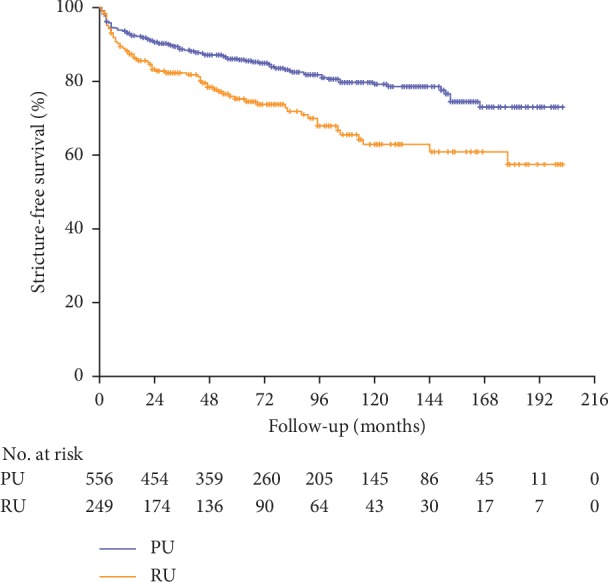
Kaplan–Meier curve for stricture-free survival in primary and redo urethroplasty. PU = primary urethroplasty; RU = redo urethroplasty; no. = numbers.

In the PU and RU groups, respectively, 95 (17%) and 68 (27%) patients suffered a failure. The 5- and estimated 10-year SFS were, respectively, 86% (95% CI: 83–89%) and 79% (95% CI: 75–83%) for the PU group and, respectively, 75% (95% CI: 69–81%) and 63% (95% CI: 55–71%) for the RU group (*p* < 0.001) (Figure 1, Table 2). Respectively, 38 (40%), 33 (35%), and 24 (25%) failures from the PU group and 29 (43%), 25 (36%), and 14 (21%) failures from the RU group occurred within the first postoperative year, between the first and fifth postoperative years and after more than five years postoperatively.

Univariate analysis identified longer strictures (HR: 1.05; *p*=0.003), multifocal strictures (HR: 2.30; *p* < 0.001), iatrogenic strictures (HR: 1.37; *p*=0.044), failed hypospadias repair (HR: 2.02; *p*=0.001), prior urethroplasty (HR: 1.78; *p* < 0.001), and diabetes (HR: 1.83; *p*=0.04) as risk factors for failure (Table 3). Prior urethroplasty (HR: 1.52; *p*=0.01) and diabetes (HR: 1.83; *p*=0.03) were identified as independent risk factors for failure in the multivariate analysis (Table 3).

## 4. Discussion

The aim of this study was to distinguish the primary and redo urethroplasty group and to compare the according SFS. Current literature shows similar success rates for primary and redo urethroplasty [7–14], although a majority of these papers indirectly compared the results of redo urethroplasty with success rates of primary procedures [8] or did not compare the results at all [7, 9, 12–14]. Since patient and stricture characteristics, definitions of failure and follow-up protocols vary among different patient series, indirect comparison is hazardous and insufficient to draw adequate conclusions. Two authors published a direct comparison between primary and redo urethroplasty, but these retrospective studies are underpowered in the amount of included redo procedures (37 and 49 respectively) [10, 11]. Hypothetically, considering 10% difference in SFS to be clinically relevant, a trial with a two-sided alpha of 0.05 and 80% power would require 248 patients per group to establish clinically relevant superiority for primary urethroplasty (assuming a SFS rate of 85% and 75% for the PU and RU group, respectively). To the best of our knowledge, this study is the first direct comparison between primary and redo urethroplasty with this amount of redo procedures and in our opinion, the results are noteworthy put the success rate of redo urethroplasty in perspective and contribute to more realistic patient expectations.

Several differences in baseline characteristics between the PU and RU groups existed. Penile strictures were significantly more frequent in the RU group, which could be explained by the fact that penile strictures are in most cases ineligible for anastomotic repair (AR) urethroplasty, which offers the highest success rate [18]. Usually, these strictures require a substitution urethroplasty and, as the success rate of these procedures deteriorates over time, the likelihood of being treated with a redo urethroplasty increases along [15, 18, 19]. Additionally, Lichen Sclerosus and failed hypospadias repair are associated with a higher failure rate and predominantly affect the penile urethra [7, 20, 21]. This in turn also explains why these etiologies were significantly more frequent in the RU group.

The redo stricture profile, as outlined above, warrants adapted operative strategies which are reflected in the applied surgical techniques [7, 20, 22]. Significantly fewer strictures were eligible for AR urethroplasty, whereas multistage procedures and definitive perineostomies were significantly more performed. This result is in line with the observations of Levine et al. [11]. Also, free graft urethroplasty was performed less frequently in the RU group. This may be explained by the fact that prior urethral surgery can impair the urethral blood supply and thus lead to a poorly vascularized, unsuitable graft bed for future urethral reconstructions. As regards the discrepancies in hospital stay between groups, patients often experience some degree of discomfort after harvesting an oral graft and this may contribute to the longer hospital stay, as observed in the PU group [23]. Meanwhile, staged procedures and perineostomies allow a relatively short hospital stay and even day surgery.

As regards surgical outcome, SFS was significantly lower in the RU group compared to the PU group, which distinctly contradicts prior literature suggesting that primary and redo urethroplasties have an equivalent outcome in terms of SFS [7–14]. However, our findings actually do corroborate the results from Blaschko et al., who described the largest redo urethroplasty series so far [7]. They reported a “primary success rate” of 67% after a median follow-up of 55 months, which is in line with our results as their definition of “primary success” corresponds with our definition of success. However, since an additional 12% of their patients remained stricture-free after the first salvage treatment, a total success rate of 78% was reported [7]. Rosenbaum et al. specifically focused on redo buccal mucosa graft urethroplasty and reported a success rate of 82% [8], albeit after a median follow-up of only 16 months, while it is established that the results of substitution urethroplasty strongly deteriorate over time [8, 19]. Siegel et al. directly compared primary and redo AR urethroplasties and described comparable results between both groups [10]. However, their sample of 37 redo procedures only contained patients with recurrent urethral strictures eligible for AR urethroplasty, representing only a favorable minority of the total patient population presenting with recurrent USD [10]. The fact that our dataset contains a mix of various techniques with different patient and stricture characteristics might explain these conflicting results. As for Levine et al., who reported the largest comparative series so far, redo urethroplasty succeeded in 92% of the cases, which was comparable with primary urethroplasty [11]. Their patient series, however, contained only 49 redo procedures and, despite the different stricture length, stricture location, and applied surgical techniques of their RU group, no Cox regression analyses were performed. Furthermore, our RU group contained substantially more penile, multifocal, panurethral, Lichen Sclerosus, and failed hypospadias repair cases which are all associated with increased stricture complexity [20–22]. Additionally, the higher success rate of Levine et al. could be explained by the fact that more than 20% of the failures of our RU group occurred after more than five years of follow-up. These failures may have been missed in their study as their mean follow-up was only 50 months [11]. Other researchers have investigated the redo urethroplasty setting as well, but their reports are characterized by a restricted sample size or a limited follow-up [9, 12–14]. Our patient series demonstrates that urethroplasty demands a prolonged follow-up since a significant amount of failures was observed after more than five years postoperatively, which is in line with the report from Han et al. [6]. An anatomical definition of failure (impossible passage of the cystoscope through the reconstructed area) could possibly detect more and earlier failures [17]. However, to date, no consensus about the definition of failure exists among urologic societies or expert panels [15].

Given the several differences in baseline characteristics between both groups, a Cox regression analysis was performed to investigate their prognostic value in the present dataset. However, in the multivariate analysis, only two characteristics remained statistically significant: prior urethroplasty and diabetes. This result underlines the observed differences in SFS between the PU and RU groups and confirms that, in this dataset, prior urethroplasty is predictive for urethroplasty failure, which corresponds with previous reports [5–7]. As for diabetes, it is known that the inherent microangiopathy contributes to a poorer vascularization, also at the urethral site, potentially impeding the healing of the urethra after surgery. This in turn can lead to an increased risk for failure [5]. Apart from these, other predictive factors for urethroplasty failure have been put forward as well, although significant differences in the investigated patient cohorts exist and might explain the inconsistent nature of these findings [3–6].

This study has various limitations. Before 2008, data were collected retrospectively and, since this cohort spans seventeen years, surgical techniques and perioperative management may have changed over time. Furthermore, every patient was offered a follow-up regimen at our institution, but a subset of patients was followed by the referring urologist, involving a risk of underreporting failures and potentially explaining a delayed detection of failures. No systematic endoscopic evaluation of urethral patency was performed and thus asymptomatic stricture formation after urethroplasty was not recorded. Also, this patient cohort represents a highly heterogeneous group involving several differences in baseline characteristics between the compared groups. The aim of this study was to explore these differences and to compare the according SFS in a patient cohort which is reflective for a tertiary reconstructive center with a minimum of exclusion criteria. Be that as it may, the aforementioned differences in SFS should be interpreted carefully, given the heterogenic nature of our comparison. However, none of the baseline characteristics, except for prior urethroplasty and diabetes, were found to be an independent risk factor for failure in the present dataset. Future studies ideally involving prospective multicenter data collection with a uniform follow-up protocol and definition of failure are required to corroborate these results in specific, more homogeneous patient subgroups and to enrich the evidence on managing recurrent USD.

## 5. Conclusions

In conclusion, several differences between primary and redo urethroplasties exist. Redo urethroplasty entails a distinct patient population to treat and is, in general, associated with lower stricture-free survival than primary urethroplasty, although more homogeneous series are required to corroborate these results. Prior urethroplasty and diabetes are independent risk factors for urethroplasty failure.

## Figures and Tables

**Table 1:** Baseline characteristics.

	Total (*n* = 805)	PU (*n* = 556)	RU (*n* = 249)	*p* value
Median age (years) (IQR)	51 (36–63)	53 (36–65)	50 (36–62)	0.3

Median follow-up (months) (IQR)	83 (46–135)	87 (50–136)	76 (40–133)	0.1

Median stricture length (cm) (IQR)	3.0 (1.5–6.0)	3.0 (1.5–6.3)	3.0 (2.0–6.0)	0.5

Stricture location *n* (%)				
Penile	207 (26)	121 (22)	86 (35)	**<0.001**
Bulbar	365 (45)	271 (49)	94 (38)	**0.005**
Posterior	102 (13)	84 (15)	18 (7.2)	**0.002**
Multifocal	63 (7.8)	41 (7.4)	22 (8.8)	0.5
Panurethral	59 (7.3)	39 (7.0)	20 (8.0)	0.7
Meatus of perineostomy	9 (1.1)	0 (0)	9 (3.6)	**<0.001**

Stricture etiology *n* (%)				
Idiopathic	276 (34)	178 (32)	98 (39)	0.050
Iatrogenic	336 (42)	247 (44)	89 (36)	**0.025**
External trauma	111 (14)	83 (15)	28 (11)	0.2
Inflammatory	73 (9.1)	41 (7.4)	32 (13)	**0.016**
Failed hypospadias repair	75 (9.3)	40 (7.2)	35 (14)	**0.004**
Tumor	9 (1.1)	7 (1.3)	2 (0.80)	0.7

Previous interventions *n* (%)				
None	170 (21)	170 (31)	0 (0)	**<0.001**
1 DVIU/dilatation	125 (16)	125 (23)	0 (0)	**<0.001**
>1 DVIU/dilatation	259 (32)	258 (46)	1 (0.40)	**<0.001**
Urethroplasty	88 (11)	0 (0)	88 (35)	**<0.001**
Urethroplasty + DVIU/dilatation	159 (20)	0 (0)	159 (64)	**<0.001**
Endoscopic realignment	3 (0.37)	2 (0.36)	1 (0.40)	>0.9
Open realignment	1 (0.12)	1 (0.18)	0 (0)	>0.9

Urethroplasty technique *n* (%)				
Transecting anastomotic repair	206 (26)	162 (29)	44 (18)	**<0.001**
Nontransecting anastomotic repair	115 (14)	91 (16)	24 (9.6)	**<0.001**
Free graft urethroplasty	264 (33)	196 (35)	68 (27)	**0.028**
Pedicled flap urethroplasty	42 (5.2)	26 (4.7)	16 (6.4)	0.3
Combined	35 (4.3)	19 (3.4)	16 (6.4)	0.1
Multistage urethroplasty	38 (4.7)	13 (2.3)	25 (10)	**<0.001**
Definitive perineostomy	43 (5.3)	17 (3.1)	26 (10)	**<0.001**
Others	62 (7.7)	32 (5.8)	30 (12)	**0.004**

Comorbidity *n* (%)				
Smoking	110 (14)	77 (15)	33 (14)	0.8
Diabetes	55 (7.1)	34 (6.4)	21 (8.7)	0.3
Cardiovascular comorbidity	138 (18)	97 (18)	41 (17)	0.7

Suprapubic catheter *n* (%)	192 (24)	147 (26)	45 (18)	**0.01**

UTI *n* (%)	216 (27)	157 (28)	59 (24)	0.2

PU = primary urethroplasty; RU = redo urethroplasty; IQR = interquartile range; cm = centimeters; DVIU = direct vision internal urethrotomy; UTI = urinary tract infection. *p* values comparing the PU group and RU group <0.05 are highlighted in bold.

**Table 2:** Pre- and postoperative characteristics.

	Total (*n* = 805)	PU (*n* = 556)	RU (*n* = 249)	*p* value
Median operation time (min) (IQR)	105 (82–131)	105 (83–130)	105 (80–135)	0.8
Median hospital stay (days) (IQR)	3 (2–4)	3 (2–4)	2 (2–4)	**0.01**
Median catheter stay (days) (IQR)	14 (10–15)	14 (11–15)	14 (9–15)	0.5
Significant extravasation at first VCUG *n* (%)	44 (7.7)	31 (7.2)	13 (8.8)	0.6
Complications (Clavien–Dindo) *n* (%)				
None	606 (75)	417 (75)	189 (76)	0.9
Grade 1	114 (14)	82 (15)	32 (13)	0.5
Grade 2	61 (7.6)	39 (7.0)	22 (8.8)	0.4
Grade 3	24 (3.0)	18 (3.2)	6 (2.4)	0.7
Stricture-free survival estimates % (SD)				
1 y-SFS		94 (1.0)	88 (2.1)	**<0.001**
2 y-SFS		91 (1.2)	83 (2.4)	
5 y-SFS		86 (1.5)	75 (3.0)	
10 y-SFS		79 (2.1)	63 (4.2)	

PU = primary urethroplasty; RU = redo urethroplasty; min = minutes; IQR = interquartile range; VCUG = voiding cystourethrography; SD = standard deviation; SFS = stricture-free survival. *p* values comparing the PU group and RU group <0.05 are highlighted in bold.

**Table 3:** Uni- and multivariate Cox regression analysis.

	Univariate		Multivariate	
	HR (95% CI)	*p* value	HR (95% CI)	*p* value
Age	1.00 (0.99–1.01)	0.7		

Stricture length	1.05 (1.02–1.08)	**0.003**	1.00 (0.95–1.06)	>0.9

Stricture location				
Penile	1.38 (0.99–1.92)	0.1		
Bulbar	0.51 (0.36–0.71)	**<0.001**	0.65 (0.42–1.01)	0.055
Posterior	0.76 (0.46–1.28)	0.3		
Multifocal	2.30 (1.47–3.58)	**<0.001**	1.71 (0.98–3.00)	0.059
Panurethral	1.45 (0.90–2.35)	0.1		

Stricture etiology				
Idiopathic	0.79 (0.56–1.11)	0.2		
Iatrogenic	1.37 (1.01–1.86)	**0.044**	1.11 (0.77–1.60)	0.6
External trauma	0.65 (0.39–1.09)	0.1		
Inflammatory	1.36 (0.85–2.18)	0.2		
Failed hypospadias repair	2.02 (1.32–4.10)	**0.001**	1.27 (0.70–2.29)	0.4

Previous interventions				
≥1 prior DVIU/dilatation	0.79 (0.58–1.07)	0.1		
≥1 prior urethroplasty	1.78 (1.30–2.43)	**<0.001**	1.52 (1.08–2.14)	**0.01**

Urethroplasty technique				
Anastomotic repair	0.46 (0.32–0.66)	**<0.001**	0.61 (0.36–1.03)	0.1
Free graft urethroplasty	1.23 (0.89–1.68)	0.2		
Pedicled flap urethroplasty	1.53 (0.88–2.66)	0.1		
Multistage urethroplasty	0.66 (0.27–1.61)	0.4		
Definitive perineostomy	1.20 (0.63–2.28)	0.6		

Comorbidity				
Smoking	1.02 (0.65–1.62)	0.9		
Diabetes	1.83 (1.09–3.08)	**0.04**	1.83 (1.07–3.11)	**0.03**
Cardiovascular comorbidity	1.22 (0.81–1.86)	0.3		

Suprapubic catheter	0.67 (0.45–1.00)	0.050		

UTI	0.91 (0.64–1.31)	0.6		

HR = hazard ratio; CI = confidence interval; DVIU = direct vision internal urethrotomy; UTI = urinary tract infection. *p* values comparing the PU group and RU group <0.05 are highlighted in bold.

## Data Availability

The data used to support the findings of this study are available from the corresponding author upon request.

## Abbreviations

AR: Anastomotic repair
CI: Confidence interval
cm: Centimeters
DVIU: Direct vision internal urethrotomy
HR: Hazard ratio
IQR: Interquartile range
min: Minutes
No.: Numbers
PU: Primary urethroplasty
RU: Redo urethroplasty
RUG: Retrograde urethrography
SD: Standard deviation
SFS: Stricture-free survival
USD: Urethral stricture disease
UTI: Urinary tract infection
VCUG: Voiding cystourethrography.
